# Smart Vape Detection in Schools for Mitigating Student E-Cigarette Use

**DOI:** 10.3390/ijerph23040501

**Published:** 2026-04-14

**Authors:** Robert Sharon, Lidia Morawska, Lindy Osborne Burton

**Affiliations:** 1 Blue IoT Pty Ltd., Mulgrave, VIC 3170, Australia; 2 ARC Training Centre for Advanced Building Systems Against Airborne Infection Transmission (THRIVE), Queensland University of Technology, 2 George Street, Brisbane, QLD 4000, Australia; lindy.burton@cdu.edu.au; 3 International Laboratory for Air Quality & Health (ILAQH), Queensland University of Technology, 2 George Street, Brisbane, QLD 4000, Australia; 4 Global Centre for Clean Air Research (GCARE), School of Sustainability, Civil and Environmental Engineering, Faculty of Engineering and Physical Sciences, University of Surrey, Guildford GU2 7XH, UK; 5 Faculty of Arts and Society, Charles Darwin University, Darwin, NT 0909, Australia

**Keywords:** indoor air quality (IAQ), adolescent vaping, vape detection, particulate matter (PM_2.5_, PM_10_), school environments, Internet of Things (IoT), real-time aerosol monitoring

## Abstract

**Highlights:**

**Public health relevance—How does this work relate to a public health issue?**

- Youth vaping has emerged as a significant public health concern, particularly in school environments where exposure to e-cigarette aerosols can occur in confined indoor spaces.
- Monitoring indoor air quality in high-risk school locations, such as restrooms, may provide new approaches for identifying and responding to vaping behaviour.

**Public health significance—Why is this work of significance to public health?**

- This study presents a real-world deployment of environmental sensors across multiple school restrooms to detect aerosol signatures associated with vaping events.
- The results demonstrate that e-cigarette use generates rapid spikes in particulate matter concentrations that can be detected using low-cost indoor air quality monitoring systems.

**Public health implications—What are the key implications or messages for practitioners, policy makers and/or researchers in public health?**

- Environmental monitoring systems may assist schools in identifying vaping activity and implementing targeted prevention or response strategies.
- Effective deployment requires integration of sensor systems with clear governance, response protocols, and stakeholder engagement to ensure that monitoring data leads to meaningful action.

**Abstract:**

Adolescent vaping has become a persistent health and behavioural challenge in schools, yet many institutions lack reliable tools to detect and respond to concealed e-cigarette use. This study addresses this problem by evaluating the real-world performance of a low-cost “Internet of Things” (IoT) vape detection system deployed across 37 high-risk restroom and change-room locations at a large Australian Independent school. The aim was to determine whether an IoT-based environmental monitoring platform could accurately identify vaping events, support timely staff intervention, and provide actionable insights into student behaviour patterns. A longitudinal case study design was used, collecting continuous particulate matter (PM_2.5_ and PM_10_) data at one-minute intervals over an 18-month period, where PM_2.5_ and PM_10_ refer to particulate matter with aerodynamic diameters ≤ 2.5 µm and ≤10 µm, respectively, reported in micrograms per cubic metre (µg/m^3^. Threshold-based alerting, cloud-based data processing, and school-led Closed-circuit television (CCTV) verification were combined to assess detection accuracy, temporal trends, and operational responses. The system recorded more than 300 vaping-related incidents, with clusters aligned to predictable times of day and higher prevalence among senior students. Operational detection performance was high, with alert events characterised by rapid, concurrent PM_2.5_ and PM_10_ excursions consistent with vaping-related aerosol profiles, although staff responsiveness declined over time due to alert fatigue and competing priorities. A major environmental smoke event demonstrated the need for context-aware logic to reduce false positives. The findings demonstrate that real-time aerosol monitoring is not only technically reliable but also highly effective in detecting vaping within school environments. These perspectives help explain why user engagement, alert fatigue, and institutional follow-through are as critical as sensor accuracy itself. Ultimately, the effectiveness of vape detection relies on strong organisational commitment, well-defined response workflows, and alignment with broader wellbeing and policy strategies. When these elements are in place, such systems can evolve from simple detection tools into intelligent, integrated components of school health governance.

## 1. Introduction

Vaping among school-aged adolescents has emerged as a significant public health challenge in Australia and across the globe. Schools represent uniquely vulnerable environments, characterised by high occupancy densities, constrained ventilation in specific spaces, and prolonged exposure durations. Electronic cigarette aerosols are dominated by fine and ultrafine particulate matter, including ultrafine particles (<0.1 µm), which are characteristic of e-cigarette emissions. While ultrafine particles are not directly measured in this study, laboratory and chamber studies demonstrate that PM_2.5_ concentrations rise sharply within seconds of vaping activity [1,2]. Complementary research has shown that poorly ventilated educational settings may also exhibit elevated volatile organic compound (VOC) levels associated with adverse respiratory outcomes in children [3], findings consistent with measurements of PM_2.5_ concentrations during e-cigarette use reaching levels comparable to conventional cigarette smoking in indoor environments [4].

Despite legal restrictions, e-cigarette use has continued to increase within secondary school settings. Evidence suggests that conventional prevention strategies, including awareness campaigns, parental engagement, and policy-based deterrence, have had limited practical influence on adolescent vaping behaviours, particularly where strong social, perceptual, and institutional factors constrain effective implementation [5,6]. In Australia, the sale or supply of vaping products to individuals under 18 years of age is prohibited, and since 1 January 2024, regulatory reforms introduced by the Therapeutic Goods Administration (TGA) have banned disposable vapes and restricted therapeutic vaping products to prescription-only pharmacy supply [7]. Nevertheless, enforcement within school contexts remains challenging, and adolescent vaping continues largely unabated, highlighting the limits of regulation alone in complex institutional environments. This gap is compounded by the limited availability of scalable, non-intrusive detection tools capable of providing timely, actionable information to support school-based prevention and response, underscoring the need for environmental monitoring approaches embedded within school governance frameworks.

The aim of this study is to evaluate the feasibility, effectiveness, and institutional implications of real-time aerosol-based vape detection in school environments, using data from an Australian independent school to assess both technical system performance and human institutional response dynamics.

A glossary of key terms, acronyms, and measurement concentrations in µg/m^3^; time in local time AET (AEST/AEDT) (units used) throughout this paper is provided in Appendix A.

By integrating technical monitoring data with qualitative and institutional analysis, this paper seeks to determine whether real-time aerosol monitoring can function as more than a reactive enforcement mechanism. Instead, it aims to position vape detection as a proactive, intelligence-led component of school health governance, capable of informing policy, supporting student wellbeing, and strengthening evidence-based risk management when embedded within clear procedural and governance frameworks.

## 2. Methods

### 2.1. Study Design and Setting

A longitudinal embedded case study design was adopted to evaluate the performance of a low-cost, IoT-enabled Long Range, low-bit-rate wireless protocol for IoT (LoRaWAN) vape detection system and to examine patterns of institutional engagement over time. The study was conducted at a large independent secondary school in Australia, occupying approximately 20 hectares and comprising more than 40 buildings, including academic blocks, sports facilities, and shared community spaces. While the Australian academic year aligns with the calendar year, the school concluded its academic term in early December and resumed in the fourth week of January 2024. During holiday periods, the campus remained partially active, with ongoing use for extracurricular sporting, community, and facility-related activities, meaning monitored environments were not entirely unoccupied. The site was selected due to its demonstrated commitment to collaborative, applied research focused on IAQ and student wellbeing. A de-identified deployment map and location index for all monitored spaces are provided in Appendix E.

This study was conducted as a single-site, in-depth case study to enable detailed observation of system performance and institutional response over an extended period. While this limits generalisability, it provides valuable insight into real-world deployment conditions and operational dynamics that may not be captured in controlled or multi-site studies.

The vape detection system was developed in partnership with an Australian IoT company and deployed across 37 high-risk restrooms and change rooms. Sensors were configured using predefined threshold levels for particulate matter (PM_2.5_ and PM_10_) and Gas-phase formaldehyde concentration, reported by the sensor in milligrams per cubic metre (mg/m^3^), as provided by the manufacturer (HCHO) to identify aerosol excursions consistent with e-cigarette emissions, which are well-established indicators of vaping activity [1,2,8]. Predefined thresholds were used to identify aerosol excursions consistent with vaping activity. Technical details of the system architecture are provided in Appendix B.

### 2.2. Sensor Selection and Parameters

Low-cost PM sensors were deployed in selected school locations to enable continuous monitoring of PM_2.5_ and PM_10_ concentrations in environments where vaping activity was most likely to occur, with emphasis on reliability, scalability, and suitability for long-term deployment [9,10]. Such sensors have been widely adopted in IAQ research as a pragmatic means of capturing high-resolution temporal variation in PM exposures where fixed reference instrumentation is impractical [9,10,11].

In addition to particulate matter measurements (PM_2.5_ and PM_10_), formaldehyde (HCHO) was monitored as the only additional parameter included in the analysis. These variables were used to support the interpretation of aerosol-generating events in high-risk locations. No additional environmental parameters (e.g., temperature or relative humidity) were incorporated into the analysis, and no external calibration or correction (including temperature or humidity adjustment) was applied beyond the manufacturer’s factory calibration.

Although the sensor platform records temperature and relative humidity, these variables were not incorporated into the event detection algorithm, and no environmental correction based on these parameters was applied. Detection performance is therefore primarily influenced by ventilation, airflow, and background particulate levels. Sensitivity is highest in confined environments with low baseline PM concentrations and limited air exchange, where transient particulate excursions are more readily distinguishable from background variability.

Devices were selected based on vendor-agnostic technical and operational criteria aligned with the requirements of distributed, low-maintenance monitoring in school environments. Key considerations included cost-effectiveness, ease of deployment, use of open communication protocols (LoRaWAN) to ensure interoperability, and cybersecurity requirements such as separation from institutional IT networks. Additional factors included battery-powered operation, suitability for long-duration monitoring, and sufficient sensitivity to detect transient particulate matter events [2,11].

Alternative devices were reviewed; however, these were not adopted due to higher cost, reliance on proprietary communication protocols, or dependence on Wi-Fi connectivity, which was considered less suitable for this deployment due to cybersecurity and network segregation requirements.

System design prioritised data security, privacy, and minimal infrastructure burden, consistent with guidance on ethically acceptable environmental monitoring in educational settings [12]. This approach also aligns with health-centred building design principles that emphasise environmental risk mitigation through building-level controls rather than individual surveillance [13]. The selected sensing approach, therefore, supported scalable, non-intrusive monitoring aligned with the study’s public health objectives while avoiding individual-level surveillance or behavioural profiling.

### 2.3. Sensor Calibration

Sensors were factory calibrated and deployed without user-adjustable calibration, consistent with established practice for low-cost optical PM monitoring.

Detailed calibration procedures and limitations are provided in Appendix D.

### 2.4. System Architecture

The vape detection system deployed in this study represents an integrated end-to-end IoT architecture, purpose-built for indoor aerosol monitoring in school environments. It leverages a LoRaWAN-based wireless sensor network, a secured cloud computing infrastructure, and real-time alerting logic to support both proactive monitoring and post-incident response.

Low-cost PM sensors were deployed in selected high-risk locations to enable continuous monitoring of indoor aerosol concentrations. Sensor data were transmitted securely via the LoRaWAN network to a cloud-based data management platform (Atrius, Acuity Brands), which enabled storage, visualisation, and structured export of time-series data for analysis. Automated logic was applied within the platform to identify rapid PM excursions consistent with vaping-related aerosol events and to notify designated staff. This design prioritised early detection and timely institutional response while avoiding individual-level surveillance or the collection of personal data.

### 2.5. Thresholds and Alert Logic

Studies indicate that vaping in confined indoor environments can result in rapid increases in particulate matter concentrations, with PM_2.5_ levels often exceeding 100 μg/m^3^ within seconds [1,8]. Based on these findings and iterative observation of sensor data patterns within the study environment, thresholds of >125 μg/m^3^ for PM_2.5_ and >175 μg/m^3^ for PM_10_ were adopted as configurable operational indicators of potential vaping events.

These thresholds were applied in conjunction with formaldehyde (HCHO) measurements to support the interpretation of aerosol-generating events. Event identification was assessed qualitatively through analysis of transient concentration spikes and comparison with contextual information, including reported incidents and CCTV observations where available. No formal statistical validation (e.g., sensitivity, specificity, or false positive rate) or controlled comparative testing of alternative aerosol sources was conducted.

**Figure 1:**
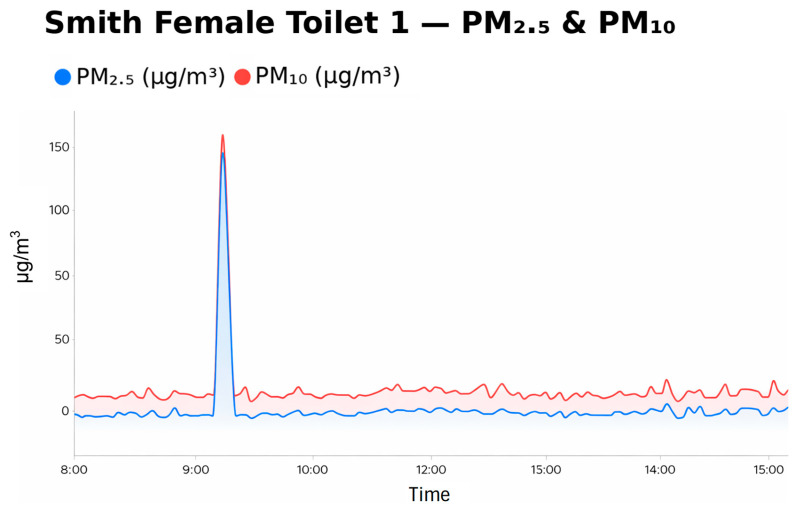
Restrooms and change rooms have minimal legitimate PM sources compared with classrooms or laboratories.

As illustrated in Figure 1 below, baseline concentrations in the study environment were typically low (PM_2.5_ ≈ 5 μg/m^3^; PM_10_ ≈ 20 μg/m^3^), with suspected vaping events characterised by rapid, short-duration spikes exceeding threshold levels, followed by decay toward baseline. In contrast, sustained elevations associated with external smoke events (e.g., backburning) exhibited prolonged high concentrations without rapid decay and were identified as a potential source of false positives. Accordingly, these thresholds should be interpreted as indicative rather than definitive markers of vaping activity.

These thresholds balance sensitivity and specificity in small, enclosed spaces such as school bathrooms.

It should be noted that the system does not chemically distinguish e-cigarette aerosol from other particulate sources, including combustion-based tobacco products. Detection is based on identifying transient particulate matter excursions consistent with known aerosol emission profiles, interpreted within environments with minimal competing PM sources. In school restroom settings, where conventional cigarette use is both prohibited and expected to be rare, and where events exhibit rapid onset and decay consistent with aerosolised emissions, detected events are interpreted as consistent with vaping rather than combustion-based smoking.

Alerts were sent to designated staff (e.g., facilities, HR officers, management) when any sensor exceeded thresholds. Staff could respond in real-time or retroactively review time-stamped CCTV footage of adjacent hallways outside. This dual-response system allowed both immediate intervention and delayed investigation, depending on resource availability.

Detailed threshold selection rationale, event logic, and tuning parameters, including environmental dependencies and system limitations, are provided in Appendix F.

### 2.6. Data Collection

Quantitative data were logged automatically at one-minute intervals per sensor and extracted monthly via the Atrius dashboard. Logged fields included timestamp, sensor identifier, PM_2.5_, PM_10_, and formaldehyde (HCHO) concentrations. Although HCHO sensing was enabled, concentrations remained at baseline levels across confirmed vaping-related events; accordingly, PM metrics were used as the primary indicators for event identification. Incident logs were cross-referenced with school-reported disciplinary outcomes for correlation analysis. This approach aligns with field-based studies indicating that particulate matter provides a more robust and reproducible signal for real-time detection of vaping-related aerosol events in occupied indoor environments.

Qualitative insights were informally collected through discussions with school staff during system walkthroughs and technical debriefs. Due to time constraints, structured interviews were not conducted, although informal themes such as “alert fatigue,” “response bottlenecks,” and “student circumvention strategies” were noted and are discussed in relation to system limitations.

A complete data dictionary defining all logged fields, units, and variables used in the analysis is provided in Appendix G, Table A2.

### 2.7. Ethical Considerations

This project was conducted with the full cooperation of school leadership. No individual students were identified, and all data were de-identified at the point of analysis. The system did not collect biometric, location, or personally identifiable information. Only environmental data were transmitted and stored. Formal ethics approval was not required under institutional policy due to the absence of human research participants; however, guidelines for secure educational data handling were followed.

This approach aligns with established ethical guidance for environmental monitoring in educational settings, where no individual-level data are collected, and no intervention targets identifiable persons [12].

Detailed cybersecurity, privacy, and data-handling controls for this deployment are described in Appendix C.

Expanded details on ethics, consent, and governance arrangements are provided in Appendix J.

## 3. Results

Data collected over the 18-month monitoring period identified more than 300 suspected vaping-related events. These events were concentrated in locations associated with senior student facilities. Cohort attribution was based on school-reported incident outcomes following investigation and disciplinary processes, rather than sensor-derived identification. As summarised in Appendix H (Table A4), the majority of identified cases involved Year 11 and Year 12 students, with only a small number of cases (*n* = 3) attributed to Year 10 students. This distribution reflects both the spatial deployment of sensors and institutional reporting practices.

Technical analysis demonstrated strong discriminative performance and temporal sensitivity, enabling reliable identification of short-duration aerosol events consistent with e-cigarette use. In contrast, operational analysis revealed constraints related to user responsiveness, alert fatigue, and variability in follow-up interventions, which moderated the system’s overall effectiveness despite continued sensor performance.

The system operated continuously between February 2023 and July 2024, generating over nine million individual data points from 37 vape detection sensors installed across restrooms and change rooms. Each sensor transmitted PM_2.5_, PM_10_, and formaldehyde (HCHO) concentrations at one-minute intervals, producing a high-resolution spatiotemporal dataset suitable for detailed event detection, trend analysis, and institutional response assessment.

Across the monitoring period, a total of over 300 aerosol events consistent with vaping were recorded. These incidents were distributed unevenly across time, buildings, and student cohorts. Table A3 (see Appendix H) presents a breakdown of incident frequencies by month and sensor location, while Table A4 (see Appendix H) includes confirmed cases correlated with student gender and year level (de-identified). Event classification was based on characteristic particulate matter profiles and contextual interpretation, with limited corroboration from staff observations and CCTV review of adjacent areas; however, individual vaping instances were not directly confirmed in all cases, and the system does not distinguish between specific aerosol sources.

Although direct confirmation of vaping behaviour was not possible, practical detection accuracy was inferred through convergent evidence. CCTV review confirmed student presence and movement patterns consistent with restroom use during alert periods, while simultaneous, rapid spikes in both PM_2.5_ and PM_10_ concentrations provided a distinctive aerosol signature unlikely to arise from normal background activity. The co-occurrence, magnitude, and temporal profile of these PM excursions support a high level of confidence that detected events reflected vaping-related aerosol emissions rather than incidental or environmental sources.

Key findings included: Year 12 students accounted for the largest number of confirmed vape incidents, followed by Year 11 and Year 10. A major event occurred on 22 March 2023, when seven female students were caught vaping simultaneously. One student was observed on CCTV vaping outside a restroom, triggering an immediate response. Despite the system’s capabilities, alert follow-up sharply declined after July 2023, attributed to competing school priorities and alert fatigue among staff.

Data analysis revealed significant temporal clustering where peak incidents occurred during lunch breaks and class transition periods. Incidents decreased in Term 4 of 2023, though alert activity remained high, suggesting a drop in staff responsiveness rather than student behaviour change. Weekly trends suggest that Mondays and Fridays exhibited higher alert frequencies, potentially reflecting both pre- and post-weekend behavioural dynamics, as well as variations in supervision and student activity patterns.

### 3.1. False Positives and External Interference

**Figure A3:**
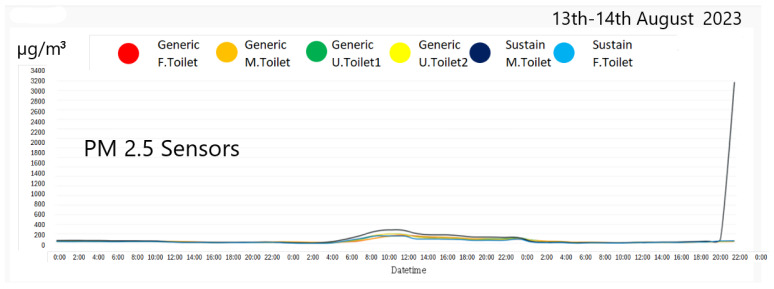
Time-series of PM concentrations during a controlled burn-off event (13–14 August 2023).

**Figure A4:**
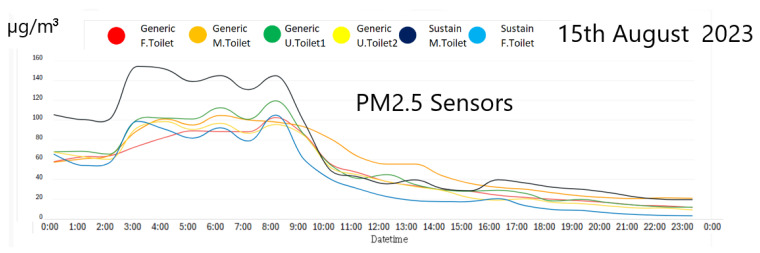
Sustained PM_2.5_ concentrations exceeding 100 µg/m^3^ across multiple monitored locations during a regional backburning event.

While system sensitivity was high, false positives were noted during regional high-pollutant events. The most prominent example occurred between 11–15 August 2023, when a controlled burn-off by local authorities introduced high levels of ambient smoke across the campus. Multiple sensors reported continuous PM_2.5_ levels exceeding 300 µg/m^3^, triggering repetitive alerts every 5 min. These alerts were false in context but valid from a sensor perspective, as the PM concentration exceeded vaping thresholds. Figure A3 and Figure A4 in Appendix I illustrate the continuous PM_2.5_ spikes across multiple toilets during this period.

This event highlighted the need for context-aware alert logic, such as cross-referencing external air quality indices, a localised outdoor PM sensor or applying temporal suppression algorithms during regional events. A detailed case study of regional smoke interference and resulting false positive alerts is presented in Appendix I.

While false positives were primarily associated with external environmental conditions, the potential for false negatives must also be considered. False negatives were not formally quantified in this study due to the absence of an independent ground-truth dataset capturing all vaping events. However, informal observations from school staff indicated that some events may not have triggered alerts, particularly where students employed strategies to reduce detectable aerosol concentrations. These included minimising exhalation volume, dispersing aerosol rapidly, or vaping in close proximity to ventilation points.

These behaviours, noted qualitatively during system walkthroughs, highlight the limitations of threshold-based detection in real-world environments and the potential for under-detection of low-intensity or deliberately concealed vaping activity. Accordingly, system outputs should be interpreted as indicative of detectable events rather than a complete record of all vaping occurrences.

### 3.2. Platform Usage and System Limitations

Despite the system’s robust architecture and alerting capabilities, several practical challenges were observed, including alert fatigue, which led to decreased responsiveness by staff after mid-2023. No students were formally disciplined for vaping after October 2023, despite continued alert activity, and a lack of formal monthly review meetings meant that data insights were underutilised, and no performance improvements were actioned post-deployment.

Additionally, formaldehyde (HCHO) concentrations remained at baseline across all detected events. In the absence of controlled laboratory or in situ validation using known vaping sources, these observations are limited to field conditions and do not allow definitive conclusions regarding formaldehyde emission characteristics or sensor sensitivity in this context. Further work should include controlled laboratory and in situ validation using representative vaping devices and e-liquids to characterise gas-phase emissions and sensor response under known conditions.

Confirmed versus unconfirmed incidents. Of all alert events, only a small subset resulted in confirmed disciplinary action. Confirmation required sensor threshold exceedance, review of CCTV entry and exit times (via the school’s internal systems), and the availability of staff to interview students. This reflects the multi-step burden of proof required in school environments and highlights the need for integrated alert workflows to streamline institutional response processes.

## 4. Discussion

Rather than focusing on laboratory characterisation or short-term pilot deployments, this study presents one of the first longitudinal, school-scale evaluations of aerosol-based vape detection integrated within real institutional workflows.

This study presents interdisciplinary research at the intersection of adolescent vaping, sensor-based detection systems, and intelligent building environments. Six thematic areas are explored: (1) adolescent vaping and school interventions, (2) vape aerosol characteristics, (3) IoT-enabled vape detection systems, (4) user-system interactions in intelligent environments, (5) policy frameworks and standards for IAQ and vaping control and (6) IAQ in Educational Institutions: Exposure and Ethics.

While the study does not measure individual health outcomes or quantify exposure-related health effects, it focuses on real-time detection of transient particulate matter excursions and institutional response as upstream public health and school-based interventions intended to reduce risk and support preventive action.

### 4.1. Adolescent Vaping and School-Based Interventions

The findings of this study reinforce existing evidence that adolescent vaping remains prevalent despite regulatory and school-based intervention efforts. The concentration of detected events in senior student areas aligns with prior research indicating higher rates of e-cigarette use among older adolescents [5,6,14]. This suggests that traditional prevention strategies may be less effective in this cohort and highlights the need for targeted approaches.

The observed reliance on environmental detection rather than behavioural reporting also reflects broader challenges identified in the literature. School-based interventions, including education campaigns and behavioural programs, have demonstrated mixed effectiveness and are often limited by resource constraints and difficulties in sustaining engagement [5,6]. In this study, qualitative observations such as alert fatigue and response bottlenecks further illustrate these operational limitations.

In this context, the deployment of infrastructure-based monitoring offers a complementary approach. The use of PM-based sensing enabled real-time identification of aerosol-generating events in environments where supervision is limited, such as restrooms and change rooms. This aligns with evidence that vaping-related emissions significantly elevate indoor particulate concentrations and contribute to degraded indoor air quality in confined spaces [1,15].

However, the findings also highlight important limitations. Detection is dependent on measurable aerosol concentrations and may be influenced by environmental conditions and user behaviour, including attempts to minimise or disperse emissions. As such, sensor-based systems should be considered as part of a broader, integrated strategy that combines environmental monitoring with education, policy enforcement, and student engagement.

### 4.2. Vape Aerosols and Detection Targets

Controlled experimental and real-world studies consistently demonstrate that e-cigarette use generates dense fine and ultrafine particulate plumes that produce sharp, high-intensity spikes in indoor PM concentrations [1,2,8]. Evaluating a range of e-cigarette devices and liquids, researchers found that vaping produces rapid PM_2.5_ and PM_10_ excursions, often reaching the hundreds of micrograms per cubic metre even under moderate puffing conditions. Vaping-related aerosol events were observed to produce transient particulate matter spikes, characterised by rapid increases followed by decay toward baseline as the aerosol plume disperses. This temporal signature provides a potential basis for distinguishing vaping events from sustained particulate matter elevations (e.g., regional smoke events); however, the alert logic in this study was based solely on threshold exceedance and did not incorporate temporal or decay-based filtering.

Across diverse settings, particulate matter concentrations measured during vaping events are substantially higher than typical indoor background levels. The magnitude of PM excursions observed in this study is consistent with prior experimental and field-based research, which reports rapid increases in PM_2.5_ concentrations during vaping activity, often exceeding several hundred μg/m^3^ [4,15]. In the present deployment, transient spikes exceeding the predefined thresholds were observed across multiple monitored locations, supporting their interpretation as aerosol-generating events consistent with vaping under real-world school conditions.

In contrast, baseline particulate matter concentrations observed in the monitored school environments (PM_2.5_ ≈ 5 μg/m^3^; PM_10_ ≈ 20 μg/m^3^) were low relative to both reported vaping-related emissions and typical indoor variability described in the literature [16,17]. This clear separation between low background levels and high-magnitude transient spikes enabled effective differentiation of aerosol-generating events within the study environment. The stability of baseline conditions across monitored locations further supported the application of threshold-based detection, as routine occupant activity did not produce comparable particulate excursions.

The concentration of events within restrooms and change rooms further supports this interpretation. These environments are characterised by minimal legitimate sources of particulate matter compared with classrooms or other occupied spaces, reducing the likelihood of confounding aerosol sources. In this study, the spatial clustering of detected events in these locations, combined with the observed temporal spike characteristics, is consistent with known emission profiles of e-cigarette use in confined indoor environments [1,8,15]. This environmental context strengthens attribution of detected particulate matter excursions to vaping-related activity rather than routine occupant movement or cleaning-related disturbances.

Although gas-phase compounds such as formaldehyde are known by-products of propylene glycol and glycerol degradation under specific operating conditions [18], real-world monitoring studies have reported inconsistent detection of these compounds in occupied indoor environments. For this reason, gas-phase sensing was retained as a secondary, exploratory parameter to support contextual interpretation rather than as a primary detection signal. Collectively, this evidence supports the selection of PM_2.5_ and PM_10_ as the primary indicators for school-based vape detection, particularly in high-use, confined spaces.

Importantly, real-time aerosol measurement studies indicate that the rate of concentration increase, rather than absolute PM concentration alone, serves as a distinguishing characteristic of vaping-related aerosol events. Rapid PM_2.5_ rises above approximately 100–300 µg/m^3^ within short time windows differentiate vaping emissions from background particulate sources such as dust resuspension or ambient infiltration [1,2,18], reinforcing the suitability of temporal, rate-based detection approaches in school restroom settings.

### 4.3. IoT-Based Vape Detection Systems

The findings of this study demonstrate the practical feasibility of deploying IoT-based air quality monitoring systems for real-time detection of aerosol-generating events in school environments. The ability to capture high-resolution temporal data across multiple distributed locations enabled the identification of transient PM spikes consistent with vaping activity, supporting the role of low-cost sensor networks in applied public health monitoring.

These results align with broader developments in IoT-enabled environmental monitoring, where low-power wireless communication protocols such as LoRaWAN enable scalable, energy-efficient deployment across large sites [19,20,21]. While such systems are increasingly adopted in commercial contexts, independent, peer-reviewed evaluations of their real-world performance in educational settings remain limited.

In this study, the use of distributed PM sensing provided a practical mechanism for identifying aerosol events in locations with limited supervision. However, system performance was influenced by environmental conditions and threshold-based logic, highlighting the importance of contextual interpretation and system tuning. These findings contribute to the limited empirical evidence based on the deployment and performance of IoT-based monitoring systems in school environments.

### 4.4. Human Factors and Intelligent Building Systems

The effectiveness of sensor-based detection in this study was closely linked to how environmental data were interpreted and acted upon by school staff. Qualitative observations, including alert fatigue and response bottlenecks, indicate that system performance cannot be assessed solely on detection capability, but must also consider institutional response capacity.

These findings are consistent with prior research demonstrating that the impact of IAQ monitoring systems depends on the usability of data interfaces, clarity of alerts, and alignment with user workflows [22,23]. In this deployment, repeated alerts during sustained pollution events (e.g., regional smoke) contributed to reduced responsiveness, illustrating the importance of contextual filtering and alert design.

The observed challenges also reflect broader findings in alarm management and risk communication, where excessive or poorly contextualised alerts can reduce user trust and engagement over time [24,25]. These results highlight the need for integrated system design approaches that combine environmental sensing with decision-support mechanisms to ensure that detected events translate into effective and sustained institutional responses.

Notably, despite the school’s strong initial commitment to IAQ research and student wellbeing, engagement with enforcement declined over time. This suggests that even in highly motivated institutional environments, sustained operational response to automated monitoring systems may be constrained by competing priorities, staff workload, and alert fatigue. This finding highlights the importance of designing systems that not only detect events, but also support sustained user engagement and actionable response over time.

In this study, alert fatigue was observed as a practical limitation, particularly during periods of repeated or non-actionable alerts such as those associated with sustained background pollution events. To address this, several technological approaches can be considered. These include threshold tuning based on site-specific baseline conditions, implementation of temporal filtering (e.g., requiring sustained exceedance or decay-based validation), and suppression of repeated alerts within defined time windows. Context-aware logic, such as integration of external air quality data or comparison with outdoor reference sensors, may further reduce false alerts during regional pollution events. Together, these approaches can improve signal relevance, reduce unnecessary notifications, and support sustained user engagement

### 4.5. Policy Frameworks and Standards

The findings of this study highlight the practical implications of the current absence of vaping-specific regulatory frameworks for school environments in Australia. In this deployment, key system parameters, including threshold selection, alert logic, and response protocols, were defined operationally rather than guided by formal standards. This reflects a broader lack of regulatory direction regarding how schools should detect, interpret, and respond to vaping-related aerosol events.

The variability observed in alert responses, including instances of alert fatigue and delayed intervention, further underscores the need for clearer policy guidance and standardised operational frameworks. Without defined benchmarks for acceptable indoor aerosol levels, event classification, or response procedures, schools are required to interpret environmental data independently, leading to inconsistent implementation and outcomes.

While broader policy approaches, such as taxation and supply restrictions, aim to reduce overall vaping prevalence [26], the findings of this study suggest that such measures alone may be insufficient to address vaping within school environments. The continued detection of aerosol events in this deployment, despite existing regulatory controls, indicates a need for complementary, infrastructure-based approaches that enable real-time monitoring and informed institutional response. These findings support the development of standardised frameworks integrating environmental monitoring with school-based policy and governance to enable consistent, evidence-based responses to vaping in educational settings.

### 4.6. System Effectiveness and Limitations

This study demonstrates that real-time environmental monitoring systems, using factory-calibrated sensors and threshold-based detection of aerosolised particulate matter, can detect aerosol events in secondary school settings. The deployed system identified aerosol events consistent with vaping, with peak PM_2.5_ concentrations often exceeding 250 μg/m^3^, substantially above typical indoor background levels and the operational thresholds applied in this study. While air quality guidelines are based on longer averaging periods, these transient peaks represent short-duration particulate matter excursions of elevated concentrations.

The system detects elevated particulate concentrations within a space and does not distinguish between active vaping and passive exposure at an individual level. As such, it reflects environmental conditions rather than attributing emissions to specific individuals or behaviours, and no direct measurement of individual exposure or dose was undertaken.

However, several limitations constrain the sustained effectiveness of such systems in practice. System effectiveness cannot be evaluated solely through technical detection performance. Despite persistent alert activity, the absence of any confirmed student consequences after October 2023 indicates a breakdown in institutional responsiveness rather than sensor capability. This pattern reflects broader evidence from school health intervention research showing that organisational capacity, competing priorities, and resource constraints frequently limit the sustained effectiveness of otherwise well-designed interventions [5,6].

In practice, sustained system impact was shaped predominantly by human, organisational, and contextual factors rather than by sensor performance alone. Alert fatigue developed over time as repeated notifications competed with staff workloads and institutional priorities, reducing responsiveness despite continued system detection performance. The requirement for multi-step verification, combining sensor alerts, CCTV review, and staff availability, introduced procedural friction that constrained timely follow-up in some cases. Environmental confounding events, particularly regional smoke episodes, also generated false positives that were technically valid but operationally unhelpful, highlighting the sensitivity of particulate-based detection to broader air quality conditions. These findings reinforce that sustained effectiveness depends less on detection accuracy than on governance alignment, workflow integration, and institutional capacity to translate alerts into action. An expanded limitations analysis and associated risk register derived from the deployment are provided in Appendix K.

No controlled pre- and post-intervention analysis of e-cigarette use was conducted in this study. While informal observations from school staff suggested increased awareness and potential deterrence following system deployment, these effects were not systematically measured or quantified. As such, behavioural impacts should be interpreted as observational and remain an area for future investigation.

### 4.7. Behavioural Engagement and Institutional Fatigue

Alert fatigue emerged as a dominant barrier to long-term system effectiveness. Staff interviews and engagement logs indicated that early enthusiasm faded within months, particularly as repeated alerts failed to result in decisive outcomes. This aligns with broader findings in risk communication literature: if alerts are not trusted, interpreted, or seen as actionable, users disengage [25,27].

Furthermore, the need for multi-layered evidence (sensor, CCTV, staff presence) placed a high operational burden on response teams. Without clear policy alignment and process simplification, even accurate alerts led to inaction or delayed intervention.

### 4.8. False Positives and Environmental Context

The 13–15 August 2023 incident, when a regional local backburning procedure caused continuous PM_2.5_ elevation across the campus, exposed a significant system design limitation. While the sensors correctly triggered alerts based on PM thresholds, the contextual misalignment (outdoor pollution vs. indoor vaping) rendered the alerts unhelpful.

This highlights the need for context-aware systems that can cross-reference regional air quality data (e.g., from the Bureau of Meteorology or outdoor sensors), temporarily suppress alerts during known events, and, where appropriate, incorporate advanced analytical approaches (e.g., pattern recognition or data-driven filtering) to support interpretation of aerosol profiles. Such enhancements are essential to ensure alert quality, reduce fatigue, and retain user trust over time.

Evaluations of low-cost PM sensors by [9,10] indicate that readings are highly sensitive to aerosol composition and environmental conditions, with non-target sources such as cleaning activities or resuspended dust frequently producing elevated signals. Without contextual interpretation, such variability can lead to misclassification of events and increased false positive rates. (see Appendix I, Figure A3 and Figure A4) illustrate the continuous PM_2.5_ spikes across multiple toilets during this period.

Potential technical refinements to mitigate such false positives include the incorporation of persistence-based logic to distinguish transient spikes from sustained particulate elevations, dynamic adjustment of alert thresholds based on background conditions, and temporary suppression of alerts during known regional pollution events. Integration with external environmental data sources, such as regional air quality indices or outdoor reference sensors, may further support context-aware alerting. These approaches would improve system specificity and reduce the frequency of non-actionable alerts in complex environmental conditions.

### 4.9. Broader Implications & Future Directions

This study reinforces a growing consensus in environmental health and socio-technical research that monitoring technologies alone are insufficient to deliver sustained behavioural or health outcomes unless they are embedded within aligned institutional practices, governance structures, and human decision-making processes. Prior research on environmental sensing and feedback systems demonstrates that the translation of data into action depends critically on interpretability, contextualisation, and organisational capacity, rather than sensor accuracy alone [23,25].

By reframing vape detection as a component of IAQ governance, rather than a narrow disciplinary or compliance mechanism, schools can address adolescent health risks in a more holistic and preventative manner. Public-health research increasingly recognises indoor environmental exposures as modifiable determinants of health in educational settings, with schools representing critical intervention environments for both behavioural risk and environmental exposure reduction [22,28,29]. Positioning vape detection within this broader IAQ framework enables schools to respond not only to individual behaviours, but to the environmental and institutional conditions that shape them.

More broadly, this work contributes to an emerging body of scholarship on data-driven harm prevention in schools, highlighting the need for closer integration between technological systems, educational practice, and public-health policy. Studies examining digital health interventions and risk communication in institutional settings suggest that meaningful impact arises when technological tools are paired with clear governance pathways, stakeholder engagement, and accountability mechanisms [12,27]. In this context, the findings of this study point toward new collaborative models between technologists, educators, and policymakers aimed at creating healthier, more adaptive learning environments.

Drawing on System-Level Enhancement Options, the empirical findings and institutional insights generated through this deployment, Table 1 below summarises a set of forward-looking options and considerations for strengthening the effectiveness, sustainability, and institutional integration of vape detection systems in school environments. These options are not intended as prescriptive recommendations, but as analytically grounded pathways that reflect the technical, governance, and human-factors dynamics observed in real-world operations.

Building on the implications and forward-looking options outlined above, further system-level enhancements were identified based on the operational performance of the deployed platform. While real-time aerosol-based vape detection was shown to be technically feasible and effective, the findings indicate that sustained impact is shaped by a combination of human, organisational, and contextual factors. In response to operational limitations observed during deployment, particularly those related to alert fatigue, user interpretation, environmental confounding, and institutional follow-through, this study identifies a set of potential technical, analytical, and user-experience enhancements. These enhancements are intended to inform future system evolution by strengthening event identification accuracy, reducing cognitive and alert burden on staff, and supporting sustained institutional engagement. A detailed operational summary of system-level technical and analytical enhancement options is provided in Appendix L (Table A6).

## 5. Conclusions

This study presents one of the earliest real-world evaluations of an IoT-enabled vape detection system deployed at scale within a secondary school environment. Using continuous PM_2.5_ and PM_10_ monitoring, LoRaWAN open systems connectivity, and automated alert logic, the system identified more than 300 vaping-related events over an 18-month period. These results demonstrate that real-time aerosol monitoring is both technically robust and operationally feasible for detecting e-cigarette use in confined, high-risk school settings.

However, the findings also reveal that technological capability alone is insufficient to guarantee sustained effectiveness. System impact was strongly mediated by institutional factors, including policy alignment, staff engagement, and response workflows. While early adoption was high, declining intervention rates emerged over time as alert fatigue and procedural misalignment reduced follow-through, even as sensor detections remained consistent. This highlights the critical importance of embedding detection technologies within a broader socio-technical ecosystem that includes clear governance structures, stakeholder training, and institutional data literacy.

Environmental context further influenced system performance. External air quality events intermittently generated false positives, underscoring the need for context-aware alert suppression, adaptive thresholds, and more advanced classification approaches. During these periods, sustained elevations in background PM concentrations rather than discrete short-duration emissions resulted in repeated alerts from affected locations. These findings reinforce the value of integrating environmental intelligence with context-aware monitoring approaches to support improved interpretation of aerosol events relative to background pollution variability.

When reframed as part of a wider IAQ governance strategy, vape detection systems can serve a broader protective function. Beyond addressing vaping behaviour, they contribute to the identification and management of environmental exposures that affect students’ cognitive performance, physical health, and psychosocial wellbeing. In this way, vape detection becomes not merely a disciplinary or compliance tool, but an intelligence-led component of school health infrastructure.

Importantly, this research reframes youth vaping in schools as an IAQ and environmental health challenge, rather than solely a behavioural issue, and demonstrates how real-time sensing can enable more responsive institutional interventions. It positions this contribution within a broader body of work advocating risk-responsive IAQ management in public and educational settings [22,28]. Through its technical deployment and empirical findings, this study advances understanding of how intelligent infrastructure can support youth vaping reduction and strengthen environmental health governance in school settings.

## Figures and Tables

**Table 1:** System-level enhancement options for vape-detection systems in school environments.

For Schools and Educational Institutions	For System Developers	For Policy and Public Health Stakeholders
Adopt Clear Response Protocols: Implement tiered response guidelines based on alert frequency and time of day. For example, repeated alerts in a single location should escalate automatically to pastoral care, not just facilities staff.	Develop Context-Aware Logic: Integrate external air quality APIs and train edge models to distinguish between vape signatures and environmental interference (e.g., smoke, deodorant).	Establish National Guidelines for vaping & smoking in Schools: Use vape detection as a gateway to broader IAQ standards.
Integrate Vaping Detection into Behaviour Policy: Align system alerts with existing wellbeing and disciplinary frameworks to ensure vaping is treated consistently with other student safety violations.	Improve Dashboard Usability: Create mobile-friendly interfaces and summary views that highlight urgent alerts without overwhelming users with raw data.	Incentivise Technology Adoption: Provide funding mechanisms for school districts to install and maintain environmental detection systems as part of public health or mental health prevention programs.
Train Staff and Communicate Expectations: Provide periodic refresher training on interpreting sensor data, accessing dashboards, and using alerts as evidence. Incorporate vaping system updates into all-staff communications.	Enable Policy Triggers: Allow schools to customise automation rules (e.g., alert thresholds, escalation logic, auto-reporting) to align with institutional workflows.	Include Vape Detection in Broader Public Health Strategies: Position vaping not just as a behavioural issue, but as an environmental exposure and social determinant addressed through integrated technological and psychosocial interventions.
Involve Students in the Solution: Promote transparency through student assemblies, classroom discussions, or peer education campaigns explaining the system’s role in promoting wellbeing, not surveillance.	Embed adaptive calibration: context-aware analytics and transparent alert logic into system design to minimise false positives, support interpretability, and enable continuous improvement based on real-world deployment data.	Support evidence-informed guidance: Frames vape detection as a preventive public health intervention, including standards for ethical deployment, data governance, response protocols, and integration with broader school wellbeing and IAQ strategies.

**Figure A1:**
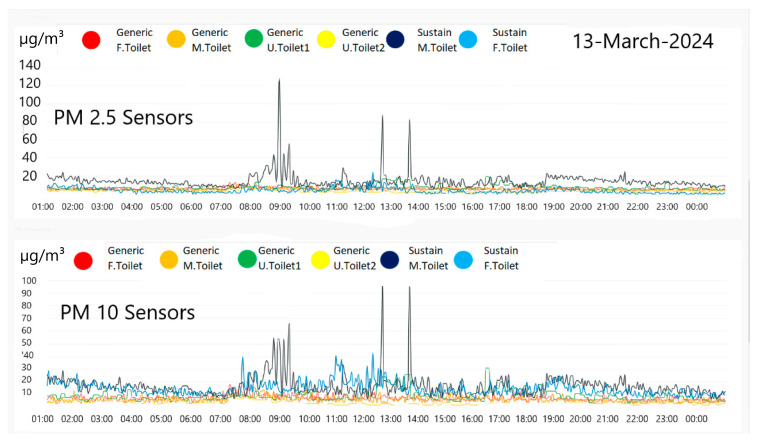
Representative time-series visualisation of PM concentrations during a suspected vaping event.

**Figure A2:**
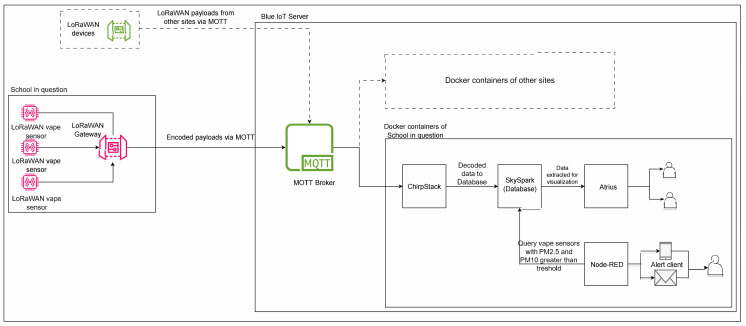
End-to-end system architecture and data flow.

## Data Availability

The datasets generated and analysed during the current study are not publicly available due to ethical, privacy, and institutional governance constraints associated with school-based environmental monitoring. De-identified and aggregated data may be made available from the corresponding author upon reasonable request, subject to approval by the participating institution and compliance with data governance and privacy requirements.

## Appendix A. Definitions, Acronyms, and Units

AQI: Air Quality Index

CCTV: Closed-circuit television

HCHO: Gas-phase formaldehyde concentration, reported by the sensor in milligrams per cubic metre (mg/m^3^), as provided by the manufacturer.

IAQ: Indoor Air Quality

IoT: Internet of Things—a network of connected sensing/actuating devices exchanging data

LoRaWAN: Long Range, low-bit-rate wireless protocol for IoT

LPWAN: Low-Power Wide-Area Network (e.g., LoRaWAN)

MQTT: Lightweight publish–subscribe protocol for telemetry

PM_2.5_: Mass concentration of airborne particulate matter with an aerodynamic diameter ≤ 2.5 µm, expressed in micrograms per cubic metre (µg/m^3^).

PM_10_: Mass concentration of airborne particulate matter with an aerodynamic diameter ≤ 10 µm, expressed in micrograms per cubic metre (µg/m^3^).

RF: Radio frequency

RBAC: Role-based access control.

Units used: Concentrations in µg/m^3^; time in local time AET (AEST/AEDT).

## Appendix B. System Architecture (Technical Details)

The vape detection system utilises a wireless open systems IoT architecture to support distributed IAQ monitoring across the school campus. Wireless communication was a fundamental requirement to minimise installation time and cost, reduce cabling requirements, and enable flexible sensor placement within existing buildings.

Communication Protocol Selection

LoRaWAN (Long Range Wide Area Network) was selected as the preferred IoT communication protocol due to its long-range capability, low power consumption, and suitability for continuous monitoring applications [20,30]. Wi-Fi was considered unsuitable due to its limited range, reliance on school-managed network infrastructure, and high power consumption when supporting frequent polling or battery-powered devices.

Protocol selection was guided by architectural suitability, security properties, and operational feasibility rather than detailed MAC-layer performance modelling, which was beyond the scope of this public-health-focused study.

Developed by Semtech in 2012, LoRaWAN is an open, low-power wide-area networking protocol designed to support scalable, multi-vendor IoT deployments. Its open-standards architecture enables interoperability between sensors and gateways from different manufacturers, reducing vendor lock-in, lowering lifecycle costs, and supporting long-term extensibility for both operational deployments and applied research [21,30]. LoRaWAN has been widely adopted for large-scale environmental and infrastructure monitoring due to its low power requirements, cost efficiency, and secure, standards-based architecture, making it well suited to distributed sensing deployments in institutional environments [30].

Cybersecurity considerations were addressed at the architectural level, focusing on protocol-level encryption, authentication, and data integrity rather than detailed threat modelling or penetration testing, which were beyond the scope of this public-health-focused study.

Network Infrastructure and Data Flow

LoRaWAN-enabled PM sensors were deployed in school toilets and change rooms and configured to transmit measurements at 60 s intervals. Sensor data packets were transmitted wirelessly to the nearest LoRaWAN gateway, which acted as a passive packet forwarder.

The school’s campus-wide infrastructure comprised seven gateways: six outdoor Milesight UG67 units and one indoor Milesight UG65 unit, providing comprehensive coverage across indoor and outdoor areas. Gateways securely relayed encrypted uplink data to the LoRaWAN network server using Lightweight publish–subscribe protocol for telemetry (MQTT) protocols over secure channels [21,30,31].

ChirpStack, hosted in a dedicated Docker container within the supplier’s cloud environment, functioned as the LoRaWAN network server. Its primary role was to authenticate devices, decode incoming payloads, and manage message routing. Once decoded, sensor data were stored in SkySpark, a specialised time-series database supporting efficient storage, retrieval, and analysis.

Alerting, Visualisation, and Hosting

To detect potential vaping activity, a Node-RED instance was configured to monitor incoming data streams against predefined PM thresholds. When thresholds were exceeded, Node-RED generated automated alerts via SMS and email to designated staff.

For visualisation and analysis, data were accessed through the Atrius (DG-Lux5) dashboard platform, allowing staff to review historical trends, examine incident timelines, and support post-incident investigations. This capability was particularly valuable when vaping was suspected but not directly observed. Figure A1 illustrates a representative time-series visualisation of PM concentrations.

All data were hosted on cloud infrastructure located at a Tier IV data centre (Micron21) in Melbourne, providing encrypted storage, high availability, and compliance with Australian cybersecurity and data-protection standards.

Figure A2 End-to-end system architecture and data flow for the deployed monitoring system, prepared for this study based on the implemented deployment, with technical input from the system provider.

System Topology and Performance Characteristics

The deployment scale, gateway density, and 60 s sampling frequency were well within documented operational limits for LoRaWAN-based environmental monitoring networks, avoiding channel saturation while supporting reliable, low-latency event detection.

Topology

Sensor (Milesight AM319) → LoRaWAN (UG67/UG65 gateways) → ChirpStack (Docker) → MQTT/RabbitMQ → SkySpark (time-series database) → Node-RED (alerting) → Atrius/DG-Lux5 (visualisation) → Email/SMS (staff)

Sampling

A total of 60 s intervals; event sampling bursts when threshold logic triggers.

Alert Logic (baseline)

- PM_2.5_ ≥ 125 µg/m^3^ or PM_10_ ≥ 175 µg/m^3^ (single breach).
- Debounce window: suppress duplicate alerts within 5–10 min for the same device.
- Escalation (optional): ≥3 alerts in 24 h at the same location → notify Pastoral/Wellbeing staff.

Reliability and Hosting

- Tier IV Micron21 data centre (Melbourne).
- TLS-encrypted transport and network segmentation.
- Reported platform uptime >99.99%.
- Gateways configured with high-gain antennas for campus coverage.

Known Limitations and Planned Upgrades (2025)

- Vendor-managed user provisioning.
- Hard-coded rule updates in the legacy platform.
- Planned enhancements include API-based AQI ingestion, machine-learning pattern matching (vape vs. smoke/deodorant), self-service alert rules, granular role-based access control, and audit trails.

## Appendix C. Cybersecurity and Privacy Controls

The vape detection system was designed with security-by-design principles to protect the confidentiality, integrity, and availability of environmental monitoring data in a school setting. Cybersecurity considerations focused on architectural safeguards, protocol-level encryption, access control, and data minimisation, rather than detailed threat modelling or penetration testing, which were beyond the scope of this public-health–focused study.

LoRaWAN Security Architecture

The system utilised the LoRaWAN protocol, which incorporates built-in security mechanisms suitable for large-scale environmental monitoring networks. Secure device activation was implemented using Over-The-Air Activation (OTAA), enabling dynamic generation of session keys for each device join. During activation, a unique Network Session Key (NwkSKey) and Application Session Key (AppSKey) are derived from a device-specific Application Key (AppKey), supporting mutual authentication between the end device and network server.

All LoRaWAN communications employed AES-128 encryption, providing separate protection for network-layer integrity and application-layer payload confidentiality. Message integrity was enforced through cryptographic Message Integrity Codes (MIC), protecting against unauthorised modification and replay attacks [21,30,31].

Transport and Cloud Security

Encrypted transport was maintained throughout the system pipeline using TLS 1.2 or higher for MQTT and HTTPS communications between gateways, network servers, and cloud-hosted services. Cloud infrastructure was segmented using virtual private cloud (VPC) controls and firewall allow-lists to restrict network access to authorised services only.

Data Minimisation and Privacy Protection

The system was designed to minimise data collection and privacy risk. Only environmental parameters relevant to vape detection were collected, including PM concentrations (PM_2.5_ and PM_10_), timestamps, device identifiers, and coded location references. No audio, video, biometric, or personally identifiable student information was collected or processed by the IoT platform.

Location identifiers were de-identified using coded room labels. Where CCTV review was undertaken, this occurred entirely within school-managed systems and was not integrated into the IoT platform, ensuring separation between environmental sensing data and student identity information.

Access Control and Auditability

User access to dashboards and alerts was restricted through named user accounts and role-based access control (RBAC), following least-privilege principles. System activity logs, including device joins, uplink messages, alert dispatch events, and user logins, were retained for operational oversight and audit purposes for defined retention periods.

Summary

Together, these measures ensured that the vape detection system operated within acceptable cybersecurity and privacy boundaries for an educational environment, supporting ethical deployment while maintaining system integrity and stakeholder trust.

## Appendix D. Sensor & Gateway Specifications

Milesight UG67 (outdoor)/UG65 (indoor) LoRaWAN/4G Gateways (Milesight Technology Co., Ltd., Xiamen, China).

- LoRaWAN packet forwarder; backhaul via Ethernet/4G.
- LoRaWAN network server: ChirpStack v3.x (most commonly v3.13.x–v3.15.x), representative of stable releases during the deployment period (2022).
- Antennas: ~6 dBi; IP67 for UG67; site-wide coverage with 6 × UG67 as supplied with the gateways.
- Firmware version: v60.x.x.x (representative of firmware available during deployment period, 2022).

(Datasheets can be attached as Appendix D.1 and Appendix D.2).

Sensor Selection and Interoperability

To meet the technical and operational requirements of this study, the Milesight AM319 was selected based on its combination of sensor accuracy, reliability, interoperability, and cost-effectiveness, attributes that are particularly well suited to large-scale deployment in school environments. Developed by Milesight, the AM319 provides multi-parameter IAQ sensing within a compact, low-power form factor designed for continuous monitoring applications.

Milesight AM319 (indoor multi-sensor) (Milesight Technology Co., Ltd., Xiamen, China)

- Available parameters: PM_2.5_, PM_10_, formaldehyde (HCHO), temperature, and relative humidity.
- Parameters collected: PM_2.5_, PM_10_, HCHO, temperature, and relative humidity.
- Parameters used in analysis: PM_2.5_, PM_10_, HCHO.

Temperature and relative humidity data were available from the sensor platform, but were not utilised in the analysis for this study.

- Range: PM 0–1000 µg/m^3^ (1 µg/m^3^ resolution); HCHO 0–1.25 mg/m^3^ (±10%).
- Power: USB (mains) for 60 s telemetry cadence
- Calibration: Factory; baseline cross-checks prior to deployment.

The AM319 utilises LoRaWAN, an open, rather than closed, low-power, long-range communication protocol that supports flexible network architectures and multi-vendor interoperability. This open-standards approach enables schools and researchers to more simply integrate sensors from any LoRaWAN-compliant manufacturer, reducing vendor lock-in, lowering lifecycle costs, and supporting long-term scalability and interoperability for both operational deployment and applied research. As such, the AM319 was well aligned with the study’s objectives of achieving technical robustness while maintaining openness, affordability, and future extensibility.

Measured Parameters and Relevance to Vaping Detection

From a functional standpoint, the AM319 incorporates a high-quality optical PM sensor and onboard formaldehyde measurement, offering direct relevance for detecting e-cigarette aerosol signatures. The device provides real-time measurements of PM_2.5_, PM_10_, temperature, humidity, CO_2_, TVOCs, and formaldehyde (HCHO), a carbonyl compound known to be generated during thermal degradation of e-liquid constituents. Laboratory studies have demonstrated that carbonyl formation increases under certain operating conditions, supporting the relevance of gas-phase sensing alongside particulate monitoring [15]. Its ability to capture simultaneous PM and gas-phase pollutants strengthens event detection accuracy, particularly in environments where students may attempt to mask vaping using aerosols, deodorants, or rapid ventilation.

Operational Suitability for School Environments

Operationally, the AM319 is designed for low-maintenance, long-duration deployments, stable factory calibration, and environmental compensation algorithms that enhance measurement reliability in restroom environments where humidity and temperature fluctuate. Competing cloud-dependent systems, especially proprietary Wi-Fi devices, typically require higher power consumption, more complex IT permissions, and more frequent calibration or servicing, making them less suited to school environments with constrained ICT support.

Taken together, these characteristics, open-protocol connectivity, multi-pollutant sensing, high reliability, low cost of ownership, and deployment ease, make the AM319 a superior choice over proprietary IAQ or vape-specific technologies, particularly for schools seeking a scalable, non-intrusive, and research-ready monitoring solution.

Factory Calibration

The Milesight AM319 incorporates a pre-calibrated optical PM sensor that is factory tested against aerosol standards under laboratory conditions. This process establishes device-specific response curves for PM_2.5_ and PM_10_ mass concentration estimates, supporting consistent baseline accuracy across units, consistent with established laboratory calibration practices for low-cost optical PM sensors [9,32].

Laser-Scattering Algorithm Calibration

The device uses a laser light-scattering method in which PM passing through an optical chamber scatters incident light that is detected by a photodiode. The resulting signal is processed using pre-programmed calibration algorithms informed by Mie scattering principles, similar to those implemented in Plantower and Cubic-class optical particle sensors widely used in low-cost IAQ monitoring [33,34]. These algorithms estimate particle size distributions and convert scattering intensity into mass concentration estimates for PM_2.5_ and PM_10_.

Environmental Compensation

As humidity and temperature influence scattering intensity and PM behaviour, the AM319 applies compensation based on its integrated temperature and relative humidity sensors. This environmental correction is consistent with the established limitations and mitigation strategies for low-cost optical PM sensors in indoor environments [33].

Long-Term Stability Measures

Long-term stability is maintained through internal firmware routines designed to monitor signal drift relative to the device’s factory baseline. Although the AM319 does not enable user calibration, automatic internal adjustments help stabilise readings over extended deployment periods, an approach common across compact optical PM sensors [9,10].

No User Calibration Required

Milesight specifies that PM_2.5_ and PM_10_ calibration is not user-accessible. Instead, the system relies on factory-calibrated optical modules and indoor environmental stability, with sensor replacement recommended if significant drift occurs. This model aligns with established practice for low-cost PM sensors used in real-time monitoring and aerosol event detection [10,35].

### Appendix D.1. Data Sheet—AM319 Sensor

https://resource.milesight.com/milesight/iot/document/am319-datasheet-en.pdf (accessed on 10 December 2025).

### Appendix D.2. Data Sheet—UG67 Gateway

https://resource.milesight.com/milesight/iot/document/ug67-datasheet-en.pdf (accessed on 10 December 2025).

## Appendix E. Deployment Map and Location Index

Campus coverage: 37 sensors across high-risk toilets/change rooms (de-identified). Proximity to gateways: ≤500 m line-of-sight typical; indoor attenuation considered in placement.

Toilet and change room locations were selected within the high school section of the campus. A total of 37 sensors were installed across the toilet and change room facilities. To protect anonymity, all building names have been replaced with fictitious identifiers, with all monitored buildings located within a 500 m radius. Table A1 summarises the deployed sensor locations and provides spatial context to support the interpretation of alert events.

**Table A1:** Sensor locations across the study site.

Description	Building Name						Grand Total
	Abacus	Generic	Harris	Evans	Sustain	Millennium	
Male Toilet/change rooms/disabled	2	6	1	6	1	1	
Female Toilet/change rooms/disabled	2	6	1	7	1	1	
Unisex toilets					2		
Total Sensors	4	12	2	13	4	2	37

## Appendix F. Thresholds, Event Logic, and Tuning

Primary trigger: PM_2.5_ ≥ 125 µg/m^3^ or PM_10_ ≥ 175 µg/m^3^ (1 min sample).

Secondary checks (optional):

- Ratio check PM_2.5_:PM_10_ > 0.6 (typical vape signature).
- Temporal pattern: rising edge ≥ 3 consecutive mins.
- Suppression: if ≥ N nearby sensors breach simultaneously → likely external smoke → suppress & banner “Regional smoke event”.

Rationale: Laboratory and field studies show that vaping events can produce PM_2.5_ peaks in the range of approximately 200–1000 μg/m^3^ in enclosed indoor environments [1,4,15]. In contrast, formaldehyde (HCHO) measurements have shown variability and inconsistency under field conditions [33,34].

Alert fatigue guardrails: Cool-down per device (5–10 min); daily digest for repeated non-critical spikes.

## Appendix G. Data Dictionary

**Table A2:** Data dictionary defining all logged fields, units, and variables used in the quantitative analysis.

Field	Type	Example	Description
timestamp_local	datetime (ISO)	2023-08-13T23:10:00 + 10:00	Local time (AEST/AEDT)
device_id	String	AM319-B01-F-T1	Unique sensor code
building_code	String	B01	De-identified building
room_code	String	F-T1	Room type/index
pm25	float (µg/m^3^)	186	1 min average
pm10	float (µg/m^3^)	214	1 min average
hcho	float (mg/m^3^)	0.00	As reported
alert_flag	Bool	1	Trigger met this minute
alert_id	String	EVT-2023-08-13-B01-001	Aggregated event key
aqi_external	Int	157	Optional external AQI
action_taken	Enum	IMMEDIATE/REVIEW/NONE	Staff response outcome

## Appendix H. Event Logs (Operational)

Table A3 presents the monthly occurrences of vaping detected in female, male, and unisex toilets, changing rooms, and showers since the system’s implementation in February 2023. The data are derived directly from the platform’s automated detection of vaping incidents.

**Table A3:** Monthly Incident Counts by Location (extract).

Areas	28-Feb-2023	31-Mar-2023	30-Apr-2023	31-May-2023	30-Jun-2023	31-Jul-2023	31-Aug-2023	30-Sep-2023	31-Oct-2023	30-Nov-2023
Female Total	14	14	4	11	10	18	8	7	21	6
Male Total	5	20	0	7	12	11	7	12	7	5
Unisex Total	0	0	0	3	1	0	5	6	1	1
Grand Total	19	34	4	21	23	29	20	25	29	12
% Change from 12 months earlier										
**Areas**	**31-Dec-2023**	**31-Jan-** **20** **24**	**29-Feb-** **20** **24**	**31-Mar-** **20** **24**	**30-Apr-** **20** **24**	**31-May-** **20** **24**	**30-Jun-** **20** **24**	**30-Jul-** **20** **24**	**28-Feb** **-2023**	**Totals**
Female Total	12	10	16	16	18	14	10	18	0	227
Male Total	14	5	5	8	2	8	1	3	0	132
Unisex Total	1	0	0	1	1	1	1	0	0	22
Grand Total	27	15	21	25	21	23	12	21		381
% Change from 12 months earlier			111%	74%	525%	110%	52%	72%		

Note: Formaldehyde was not detected during confirmed events in this deployment.

Table A4 was compiled using data provided by the school. To protect the school’s identity, several building locations have been anonymised. The table records each vaping incident, including the date, time, PM_2.5_ and PM_10_ readings, number of students involved, gender, and school year. Year 12 students accounted for the majority of incidents, followed by Year 11 and a smaller number from Year 10. The most notable event occurred on 22 March 2023, when seven female students were caught simultaneously. Remarkably, one Year 11 student was observed vaping openly in the common walkway outside the toilets and was captured on CCTV.

**Table A4:** Confirmed Incidents (School-verified; de-identified).

Date	Time	Location	Number of Students	M/F	School Year	PM_2.5_ Reading	PM_10_ Reading	Notes
6 March 2023	08:34 a.m.	Girls’ Shower/Toilet/changing room	2	F	10	161	242	
8 March 2023	08:26 a.m.	Girls’ Shower/Toilet/changing room	1	F	11			
8 March 2023	08:21 a.m.	Girls’ Shower/Toilet/changing room	2	F	12	116	122	
8 March 2023	08:33 a.m.	Girls’ Shower/Toilet/changing room	3	F	11	152	226	*** Vape seen in hand CCTV
9 March 2023	08:33 a.m.	Boys’ Shower/Toilet/changing room	1	M	12	327	519	
10 March 2023	2:45 p.m.	Boys’ Shower/Toilet/changing room	2	M	11			
22 March 2023	08:33 a.m.	Boys’ Shower/Toilet/changing room	1 × Parent	M	Adult	153	157	
22 March 2023	08:33 a.m.	Girls’ Shower/Toilet/changing room	7	F	11	109	178	*** Vape seen in hand CCTV
22 March 2023	08:47 a.m.	Boys’ Shower/Toilet/changing room	1	M	11	85	130	
24 March 2023	1:02 p.m.	Boys’ Shower/Toilet/changing room	1	M	12	215	254	
24 March 2023	8:32 a.m.	Boys’ Shower/Toilet/changing room	1	M	12	141	211	
26 March 2023	04:32 p.m.	Boys’ Shower/Toilet/changing room	2	M	12	65	93	
28 March 2023	6:47 p.m.	Boys’ Shower/Toilet/changing room	1 × Parent	M	Adult	1047	1222	
28 March 2023	12:47 p.m.	Boys’ Shower/Toilet/changing room	1	M	12	174	230	
29 March 2023	10:32 a.m.	Girls’ Shower/Toilet/changing room	1	F	12	140	192	
30 March 2023	2:47 p.m.	Boys’ Shower/Toilet/changing room	1	M	12	161	187	
30 March 2023	11:18 a.m.	Boys’ Shower/Toilet/changing room	1	M	12	170	228	
30 March 2023	9:17 a.m.	Girls’ Shower/Toilet/changing room	2	F	11	150	372	
18 April 2023	3:33 p.m.	Girls’ Shower/Toilet/changing room	1	F	12	160	253	
19 April 2023	8:33 a.m.	Girls’ Shower/Toilet/changing room	3	F	12	131	199	
26 April 2023	9:34 a.m.	Girls’ Shower/Toilet/changing room	1	F	12	170	175	
2 May 2023	8:32 a.m.	Girls’ Shower/Toilet/changing room	4	F	12	138	224	
3 May 2023	08:32 a.m.	Girls’ Shower/Toilet/changing room	5	F	11	178	279	
5 May 2023	3:47 p.m.	Girls’ Shower/Toilet/changing room	1	F	12	200	249	
8 May 2023	12:32 p.m.	Girls’ Shower/Toilet/changing room	3	F	11	129	197	
9 May 2023	10:20 a.m.	Girls’ Shower/Toilet/changing room	1	F	12	237	254	
12 May 2023	1:02 p.m.	Girls’ Shower/Toilet/changing room	3	F	12	287	467	
15 May 2023	1:17 p.m.	Boys’ Shower/Toilet/changing room	1 × Teacher	M	Adult			
17 May 2023	8:22 a.m.	Girls’ Shower/Toilet/changing room	1	F	12	71	91	
17 May 2023	8:22 a.m.	Girls’ Shower/Toilet/changing room	2	F	11	165	239	
23 May 2023	3:32 p.m.	Girls’ Shower/Toilet/changing room	1	F	11	82	124	
25 May 2023	10:47 a.m.	Disabled Toilets	1	M	10	165	181	
11 June 2023	11:02 a.m.	Girls’ Shower/Toilet/changing room	2	F	11	136	204	
12 July 2023	8:38 a.m.	Girls’ Shower/Toilet/changing room	1	F	11	471	518	
13 July 2023	11:23 a.m.	Girls’ Shower/Toilet/changing room	1	F	12	254	267	
18 July 2023	8:22 a.m.	Boys’ Shower/Toilet/changing room	1	M	11	327	355	
19 July 2023	1:37 p.m.	Boys’ Shower/Toilet/changing room	1	M	12			
27 July 2023	3:48 p.m.	Girls’ Shower/Toilet/changing room	1	F	12	381	441	

## Appendix I. False Positives & External Events (Case Study)

Controlled burn-off (11–15 August 2023)

Figure A3 below is dated from the 13th to the 14th of August 2023. The alerts started late on the 13th and then again late on the 14th. In fact, the sensor in this location went through its limit.

Figure A4 shows the sustained PM_2.5_ concentrations exceeding 100 µg/m^3^ across multiple monitored locations during the regional backburning event.

## Appendix J. Ethics, Consent, and Governance

- Human data: Not collected; environmental signals only.
- Consent: School community notice about IAQ monitoring for health/safety; no student tracking.
- Governance: Oversight by School Leadership & Facilities; periodic review with vendor; data retention 24 months; deletion on request per policy.
- Template: Community Notice (short form)
- “Our school monitors IAQ in selected amenities to deter e-cigarette use and protect health. Sensors measure particles (PM_2.5_/PM_10_) only; no cameras or audio are used. Data are de-identified and used for safety, wellbeing, and facility management.”

## Appendix K. Limitations and Risk Register (Expanded)

**Table A5:** Expanded limitations and risk register for the deployed vape-detection system.

Risk	Description	Likelihood	Impact	Mitigation
External smoke	Regional burns/bushfires elevate PM	Medium	High	AQI integration; campus-wide correlation; suppression
Alert fatigue	Repeated alerts reduce responsiveness	High	High	Cool-downs; escalations; monthly reviews
User provisioning	Vendor-managed onboarding	Medium	Medium	Next-gen self-service admin
Sensor drift	LCS accuracy over time	Medium	Medium	QA/QC schedule; swap-outs
Power loss	USB power disruption	Low	Medium	UPS at outlets; device offline alerts

## Appendix L. System-Level Enhancement Options

Table A6 outlines potential system-level enhancement options, including technical, operational, and workflow-related improvements identified during the deployment.

**Table A6:** System-level enhancement options spanning technical, operational, and workflow considerations.

Proposed Upgrade	Description	Operational Limitation Addressed (Study Finding)	Expected Benefit
Enhanced dashboards	Redesigned UI with trend visualisation, daily summaries, and comparative room analysis	Dashboard misinterpretation and high cognitive load reduced staff responsiveness	Improves data interpretability, reduces cognitive burden, and supports timely decision-making
User-defined dashboards	Customisable dashboards tailored to user roles (facilities, wellbeing, leadership)	Generic dashboards lacked contextual relevance for different stakeholder groups	Increases sustained engagement and aligns insights with user responsibilities
Regional air quality integration	API-based cross-referencing with government ambient AQI data	External pollution events caused temporary false positives	Suppresses false positives and improves confidence in alerts
Outdoor contextual sensing	Deployment of outdoor PM_2.5_, PM_10_, and TVOC sensors	Lack of environmental context limited indoor event interpretation	Provides site-level context and improves event classification accuracy
Anthropic AI model analytics (cloud + edge)	Cloud-based large language models combined with lightweight edge AI trained on particulate patterns	Difficulty distinguishing vaping from confounding aerosol sources (e.g., deodorants, smoke)	Reduces alert fatigue and increases classification precision
Policy-linked alerts	Embedded escalation logic aligned with school wellbeing policies	Alerts did not consistently trigger structured follow-up actions	Ensures consistent, policy-aligned responses and institutional follow- through
Monthly review automation	Automated monthly reports on incidents, trends, and response rates	Lack of routine reflection and governance-level oversight	Encourages accountability, learning, and evidence-based governance
Alert confidence scoring	Assigns probabilistic confidence levels to alerts based on signal strength, rate of rise, and duration	Binary alerts contributed to alert fatigue and difficulty prioritising responses	Improves trust in alerts, prioritisation, and response quality
Temporal alert suppression	Implements cool-down logic to suppress repeated alerts from the same location within a defined time window	Multiple alerts triggered by single events increased cognitive load	Reduces redundant notifications and alert fatigue
Response capture and tagging	Allows staff to classify alert outcomes (e.g., verified event, false positive, no action)	Lack of feedback limited learning and system refinement	Enables response-rate analytics and improves AI training datasets
Embedded staff training module	Short, role-specific onboarding explaining alert logic, interpretation, and actions	Limited user training reduced effective system use	Improves interpretation, confidence, and institutional uptake
Ventilation correlation analytics	Correlates aerosol persistence with ventilation status or inferred decay rates	Limited insight into environmental drivers beyond behaviour detection	Strengthens IAQ governance and health-focused outcomes
Privacy-preserving analytics	Ensures analytics focus on locations and temporal patterns without individual attribution	Ethical and policy concerns risk limiting acceptance	Improves trust, compliance, and long-term adoption
System health and sensor drift monitoring	Automated diagnostics for sensor uptime, drift detection, and data quality flags	Undetected sensor degradation risks silent performance failure	Maintains long-term reliability and confidence in the system
